# Proteomic analysis of purified protein derivative of *Mycobacterium tuberculosis*

**DOI:** 10.1186/1559-0275-10-8

**Published:** 2013-07-19

**Authors:** Thottethodi Subrahmanya Keshava Prasad, Renu Verma, Satish Kumar, Raja Sekhar Nirujogi, Gajanan J Sathe, Anil K Madugundu, Jyoti Sharma, Vinuth N Puttamallesh, Anjali Ganjiwale, Vithal P Myneedu, Aditi Chatterjee, Akhilesh Pandey, HC Harsha, Jayasuryan Narayana

**Affiliations:** 1 Institute of Bioinformatics, International Tech Park, Whitefield, Bangalore 560 066, India; 2 Centre for Bioinformatics, School of Life Sciences, Pondicherry University, Puducherry 605 014, India; 3 Manipal University, Madhav Nagar, Manipal 576 104, India; 4 School of Biotechnology, KIIT University, Bhubaneswar, Odisha-751024, India; 5 Microtest Innovations Pvt. Ltd., International Tech Park, Whitefield, Bangalore 560 066, India; 6 Armed Forces Medical College, Pune 411 040, India; 7 Department of Microbiology, LRS Institute of TB and RD, New Delhi 110 030, India; 8 Johns Hopkins University School of Medicine, Baltimore, MD 21205, USA

**Keywords:** Biomarker, Broad spectrum antibiotics, Epitope, LC-MS/MS, Mantoux test

## Abstract

**Background:**

Purified protein derivative (PPD) has been used for more than half a century as an antigen for the diagnosis of tuberculosis infection based on delayed type hypersensitivity. Although designated as “purified,” in reality, the composition of PPD is highly complex and remains ill-defined. In this report, high resolution mass spectrometry was applied to understand the complexity of its constituent components. A comparative proteomic analysis of various PPD preparations and their functional characterization is likely to help in short-listing the relevant antigens required to prepare a less complex and more potent reagent for diagnostic purposes.

**Results:**

Proteomic analysis of Connaught Tuberculin 68 (PPD-CT68), a tuberculin preparation generated from *M. tuberculosis*, was carried out in this study. PPD-CT68 is the protein component of a commercially available tuberculin preparation, Tubersol, which is used for tuberculin skin testing. Using a high resolution LTQ-Orbitrap Velos mass spectrometer, we identified 265 different proteins. The identified proteins were compared with those identified from PPD *M. bovis,* PPD *M. avium* and PPD-S2 from previous mass spectrometry-based studies. In all, 142 proteins were found to be shared between PPD-CT68 and PPD-S2 preparations. Out of the 354 proteins from *M. tuberculosis*–derived PPDs (i.e. proteins in either PPD-CT68 or PPD-S2), 37 proteins were found to be shared with *M. avium* PPD and 80 were shared with *M. bovis* PPD. Alignment of PPD-CT68 proteins with proteins encoded by 24 lung infecting bacteria revealed a number of similar proteins (206 bacterial proteins shared epitopes with 47 PPD-CT68 proteins), which could potentially be involved in causing cross-reactivity. The data have been deposited to the ProteomeXchange with identifier PXD000377.

**Conclusions:**

Proteomic and bioinformatics analysis of different PPD preparations revealed commonly and differentially represented proteins. This information could help in delineating the relevant antigens represented in various PPDs, which could further lead to development of a lesser complex and better defined skin test antigen with a higher specificity and sensitivity.

## Background

Around 2 billion people in the world are infected with *M. tuberculosis*. According to WHO world TB (tuberculosis) control report 2011, in 2010 alone, 9 million new TB cases were reported and 1.45 million deaths occurred worldwide. Tuberculosis is the second most common infectious killer disease after HIV. One in five of the 1.8 million AIDS related deaths are estimated to be associated with TB. Tuberculin skin test (TST) is the standard test for the diagnosis of TB infection in the Western world [1]. American Thoracic Society and Center for Disease Control and Prevention recommend targeted TST for deciding the treatment regimen among groups associated with increased risk for progression of latent tuberculosis infection (LTBI) to active TB [2]. Vaccination is an important preventive measure to control community load of TB. An attenuated strain of *M. bovis* known as Bacillus Calmette-Guerin (BCG) is universally employed as a vaccine against TB. However, efficacy of BCG is controversial as it does not protect against adult forms of pulmonary tuberculosis [3,4]. Moreover, prior exposure of individual to environmental mycobacteria and organisms sharing antigenic epitopes results in recall of immune memory response to BCG administration [3]. After almost 12 decades of research, we still do not have a reliable diagnostic test for TB that can be used in primary health care centers with definitive results.

In 1890, Robert Koch introduced boiled, crude extract of tubercle bacilli in glycerin (referred as “old tuberculin”) as a potential vaccine material against tuberculosis infection [5-7]. Although Koch’s old tuberculin could not be used as therapy because of its toxicity, impurity and inadequate standardization; the concept of tuberculin was instrumental in laying the foundation of the modern TST [8]. TST, first introduced by Von Pirquet in 1909 [6] has been in use as a standard method for diagnosing TB infection almost over the last six decades [8,9]. It is based on measuring the extent of induration formed because of delayed type hypersensitivity reaction elicited by mycobacterial antigens present in PPD.

In addition to its role in detecting mycobacterial infection, TST has also been used as a standard tool to estimate the prevalence of LTBI [8]. The role of PPD in serodiagnosis of TB, with sensitivity as high as 92%, was reported in Warao and Creole populations [10]. Several studies reported the use of PPD in serodiagnosis of tuberculosis infection with high sensitivity [11,12]. PPD has also been used as a standard control in immunological assays [13]. It is reported that PPD improves the sensitivity of interferon gamma release assay (IGRA). IGRA uses early secretory antigenic target-6 (ESAT-6) and culture filtrate antigen EsxB (CFP10) antigens present in *M. tuberculosis* and *M. bovis* but not in BCG. This can enable differentiation of TB-infected and BCG vaccinated individuals [14,15]. However, Yassin et al. reported that sensitivity of IGRA can be compromised in children with severe malnutrition and HIV co-infection. Concomitant use of TST, IGRA and interferon gamma induced protein 10 (IP-10) in children staying in contact with smear-positive adults has shown higher number of children as positive [16]. In addition, IGRAs suffer from limitations including higher cost, variable sensitivity, poor reproducibility, limited interpretive criteria and unknown prognostic value [17]. Despite its important applications, PPD is not considered as a reliable material. This is due to high rates of false positive results, inability to distinguish between tuberculous and non-tuberculous mycobacteria or individuals vaccinated with BCG [18]. This can be attributed to immune response elicited by antigens from BCG or environmental bacteria sharing antigenic epitopes [19,20]. Earlier studies by Borsuk et al. identified molecular chaperone DnaK (DnaK), molecular chaperone GroEL (GroEL2), elongation factor 2 (EF-Tu), cell surface lipoprotein Mpt83 (Mpt83) and acyl carrier protein as abundant proteins common to *M. bovis* and *M. avium* PPDs [21]. Moreover, discrepancy of results has been observed between different PPD preparations [22,23]. Currently available PPD preparations used on human subjects include PPD-S2 [6], PPD-RT23 [24], PPD IC-65 [9,13] and PPD-CT68 [25].

Knowledge about the constituents of PPD could allow the researchers to effectively work on PPD associated diagnostic complications. Earlier studies employed gel electrophoresis to identify constituents of PPD [26]. Kuwabara and Tsumita in 1974 first attempted to identify and characterize the components of PPD [27]. An analysis which employed gel electrophoresis for characterization of PPD antigens in whole cell lysate of *M. bovis* BCG resulted in four protein bands corresponding to PPD [28]. Kitaura et al. could distinctly identify only two ribosomal proteins L7 and L12 in *M. tuberculosis* and *M. bovis* PPDs in gel electrophoresis [29]. With the advent of high resolution mass spectrometry, it is now possible to identify proteins from complex peptide mixtures. Borsuk et al. identified 171 proteins in an LC-MS/MS analysis of Brazilian and UK bovine and avium PPDs [19]. Cho et al. recently identified 240 proteins in PPD-S2 [26]. PPD-CT68, which is another standard reagent used for TST, has not been analyzed thus far. In the present report, we have analyzed and described the proteome profile of PPD-CT68 using high resolution mass spectrometry and compared it with that of other PPDs derived from *M. tuberculosis, M. avium* and *M. Bovis*. PPD-CT68 examined here was developed by Landi in 1963 from “Johnston” strain of *M. tuberculosis* var. *hominis*[30].

## Results and discussion

### Identification of proteins present in PPD-CT68 from *Mycobacterium tuberculosis*

**Figure 1:**
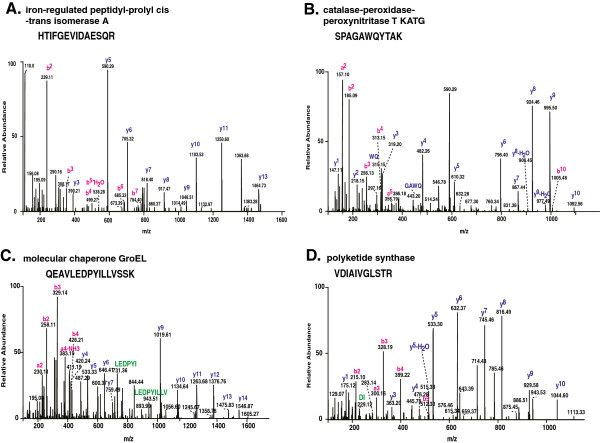
**MS/MS spectra of peptides from some of the *M. tuberculosis *PPD-CT68 proteins identified in this study (A) iron-regulated peptidyl-prolyl cis-trans isomerase A (B) catalase-peroxidase peroxynitritase T KatG (C) molecular chaperone GroEL (D) polyketide synthase.**

We carried out a proteomic profiling of PPD-CT68 prepared from *M. tuberculosis* culture in a protein-free medium using high resolution Fourier transform mass spectrometry. Mass spectrometry-derived data was searched using Sequest algorithm embedded in the Proteome Discoverer software against a protein database of *M. tuberculosis* from NCBI RefSeq. Search of 5,205 MS/MS spectra resulted in 1,146 peptide-spectrum matches, which corresponded to 695 unique peptides. The list of peptides identified in this study is provided in Additional file 1: Table S1. Representative MS/MS spectra are provided in Figure 1. Based on these 695 unique peptides, we identified 265 proteins (Additional file 2: Table S2) of *M. tuberculosis* in PPD-CT68.

**Figure 2:**
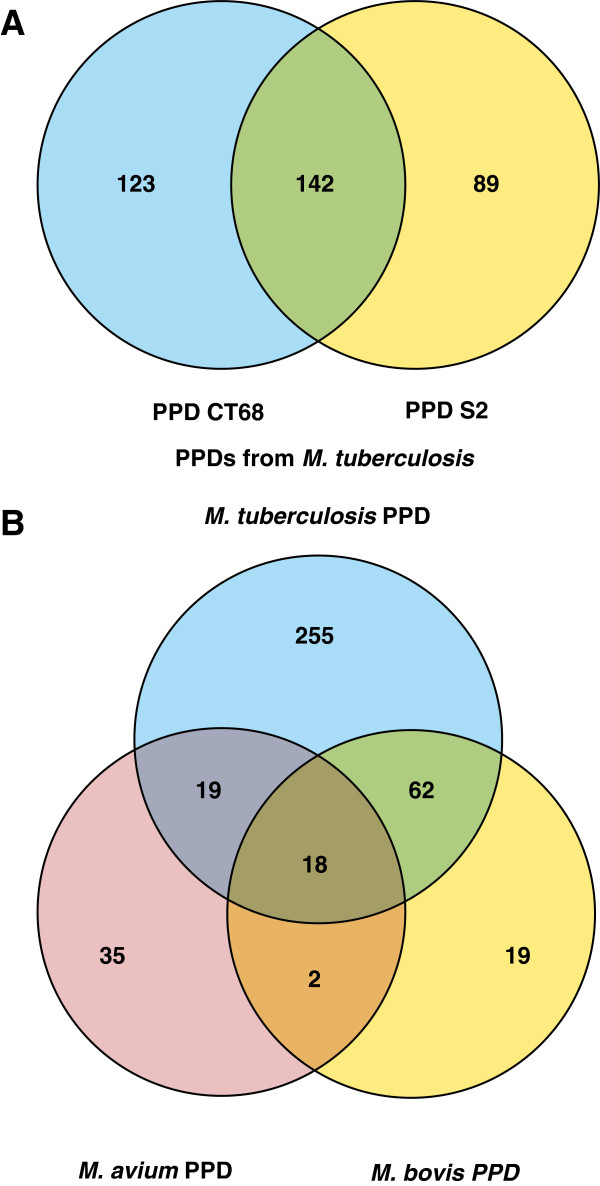
**Overlap of proteins identified among PPD-CT68 and PPD-S2 (A) Proteins identified among *M. tuberculosis *PPD, *M. avium *PPD and *M. bovis *PPD (B).**

Cho and colleagues [26] recently reported the identification of 240 proteins from PPD-S2, which is the standard for TST, as recommended by U.S. Food and Drug Administration (FDA). Out of 240 proteins listed, 231 are non-redundant. We compared proteomic results obtained in our study with proteins identified from PPD-S2 (Figure 2A). Out of the 265 proteins identified from PPD-CT68, 142 proteins were shared with PPD-S2, whereas, 123 and 89 proteins were exclusively identified from PPD-CT68 and PPD-S2, respectively. Altogether, 354 proteins of *M. tuberculosis* PPD have been identified.

For further understanding of protein profiles of various PPD preparations, we compared the PPD derived from *M. tuberculosis* (PPD-CT68 and PPD-S2) with PPDs of *M. bovis* and *M. avium*[19] (Figure 2B). Out of 354 proteins from *M. tuberculosis* PPDs, 37 proteins were found common with *M. avium* PPD and 80 were common with *M. bovis* PPD. We also found that 18 proteins were common in PPDs obtained from *M. tuberculosis*, *M. bovis* and *M. avium*. When compared to PPDs from *M. tuberculosis*, 35 and 19 proteins were exclusively found in *M. avium* and *M. bovis* PPDs respectively. It is also interesting to note that 255 proteins were exclusively identified in PPDs from *M. tuberculosis*.

### Functional analysis of proteins common among all PPDs

We carried out functional classification of proteins identified in *M. tuberculosis* (PPD-CT68 and PPD-S2), which are also present in *M. avium* and *M. bovis*. Most of the proteins were implicated in causing infection and protecting the pathogen against various metabolic stresses. Five of eighteen proteins- secreted antigen 85A (FbpA), thiol peroxidase (Tpx), bacterioferritin (BfrA), thioredoxin (TrxC) and lipoprotein LprG (LprG) - offer protection against oxidative and nitrosative stress. On the other hand, co-chaperonin GroES (GroES), DnaK, serine protease PepA (PepA), alanine and proline rich secreted protein Apa (Apa) and hypothetical protein Mpt64 are involved in causing infection. Detailed functional classification of each protein is given in the Table 1.

**Table 1:** Functional classification of proteins common in all PPDs

	**Gene symbol**	**Description**	**Function**	**Reference**
1	AcpP	Acyl carrier protein	It shuttles the intermediates between the enzymes of type II fatty acid synthase system	[31]
2	FbpA	Secreted antigen 85-A	It participates in cell wall biosynthesis, and interacts with the host macrophage as fibronectin-binding protein. It is also involved in establishment and maintenance of a persistent tuberculosis infection	[32,33]
3	GroES	Co-chaperonin	It is a dominant immunogenic protein	[34]
4	DnaK	Molecular chaperone	It is highly antigenic and act as co-repressors for heat shock protein transcriptional repressor (hspR)	[35]
5	Tpx	Thiol peroxidase	It protects *M. tuberculosis* against oxidative and nitrosative stress	[36]
6	RpiL	50S ribosomal protein L7/L12	It is involved in interaction with translation factors	[37]
7	BfrA	Bacterioferritin	It is an intracellular iron storage protein, which protects Mycobacterium from oxidative stress mediated by excess iron	[38]
8	SahH	S-adenosyl-L-homocysteine hydrolase	It is a ubiquitous enzyme that plays a central role in methylation-based processes by maintaining the intracellular balance between S-adenosylhomocysteine (SAH) and S-adenosylmethionine	[39]
9	TrxC	Thioredoxin	It is involved in redox homeostasis and uses it to protect the pathoen against the oxidative intermediates generated by the macrophages	[40]
10	FixB	Electron transfer flavoprotein subunit alpha	It is electron transfer flavoprotein subunit alpha, in some bacteria it functions as nitrogen fixing agent but its function in *M .tuberculosis* is not clear	[41]
11	PepA	Serine protease	It is a serine protease associated with cell membrane, which stimulates peripheral blood mononuclear cells from healthy PPD donors to proliferate and secrete gamma interferon	[42]
12	Wag31	Hypothetical protein	It is a cell division initiation protein involved in regulation of genes including virulence factors and antigens	[43]
13	Mpt64	Hypothetical protein	It is an immunogenic protein which elicits delayed type hypersensitivity skin response	[44]
14	Apa	Alanine and proline rich secreted protein	It is a cell surface glycoprotein which binds to host lectins and cheat the innate immune system	[45]
15	LprG	Lipoprotein LprG	It plays a role in *M. tuberculosis* infection by inducing increased suppression of the immune response due to elevated nitric oxide production	[46]
16	Rv1893	Hypothetical protein	Function unknown	[47]
17	Rv1855c	Oxidoreductase	Probable monooxygenase	[48]
18	Gap	Glyceraldehyde-3-phosphate dehydrogenase	It has glyceraldehyde-3-phosphate dehydrogenase activity	[49]

### Bioinformatics analysis of PPD-CT68 proteins showing homology to lung infecting bacteria

**Figure 3:**
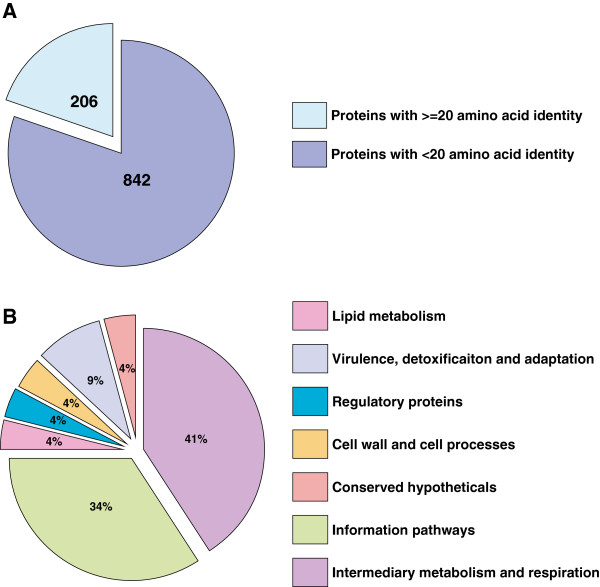
**Pie chart representing lung infecting bacterial proteins with <20 amino acid long peptides and ≥20 amino acid long peptides identical to PPD-CT68 proteins (A). **Functional classification of 47 PPD-CT68 proteins showing identity of 20 or more amino acids in 24 lung infection associated bacterial proteins **(B)**.

Raman et al. performed a comprehensive analysis on *M. tuberculosis* genes homologous to 228 different pathogenic bacteria [50]. We further analyzed 265 proteins represented in PPD-CT68 against the proteins encoded by 24 lung infecting bacteria selected from the list of 228 pathogens. Protein BLAST was performed to locate regions of PPD-CT68 proteins sharing 10 or more identical amino acid long regions with bacterial proteins. In all, 3,446 peptides from 24 pathogens corresponding to 1,048 proteins showed 10 or more amino acid identity with 117 proteins from PPD-CT68 (Additional file 3: Table S3a and S3b). Since, a peptide of 20 or more amino acid residues can be a potential epitope, we further shortlisted the proteins reflecting identity in a continuous stretch of 20 or more amino acids (Additional file 3: Table S3c). Two hundred and six out of 1,048 bacterial proteins showed identity with 47 PPD-CT68 proteins. (Figure 3A). Functional analysis of 47 mycobacterial proteins sharing identical regions revealed that 41% proteins are associated with intermediary metabolism and respiration, 34% with information pathways, 9% with virulence and detoxification, 4% with cell wall and cell processes, 4% with lipid metabolism and 4% with regulatory proteins (Figure 3B).

To further study the role of these 47 proteins in DTH and serodiagnosis, we compared our data with immunoproteome of *M. tuberculosis* reported by Velayudhan et al., which included 484 mycobacterial proteins recognized by human sera collected from worldwide TB suspects [51]. Thirteen proteins - DNA-directed RNA polymerase subunit beta; DNA-directed RNA polymerase subunit alpha; GroEL; 30S ribosomal protein S1; fumarate hydratase; elongation factor G; DnaK; aconitate hydratase; isocitrate dehydrogenase; S-adenosyl-L-homocysteine hydrolase; malate synthase G; D-3-phosphoglycerate dehydrogenase; and enoyl-CoA hydratase - were recognized by antibodies in serum. Out of these 13 proteins, only seven (isocitrate dehydrogenase; malate synthase G; succinyl-CoA synthetase subunit alpha; malate dehydrogenase; succinyl-CoA synthetase subunit beta; aconitate hydratase; and type II citrate synthase) were listed in immunoproteome and were identified in our mass spectrometry analysis of PPD-CT68 [51]. These proteins are associated with a host immune response in cases with active tuberculosis.

### PPD proteins as candidate biomarkers

Available knowledge of the *M. tuberculosis* genes provides us the advantage to express and synthesize recombinant purified antigens, which gives us the opportunity to test new biomarkers for TB infection. These antigens can be used to detect the antibodies in the serum and have a potential to improve diagnosis. Several studies have explored the use of the recombinantly expressed antigens and evaluated their immunodiagnostic potential. An updated systematic review on the diagnostic accuracy of commercial serological tests for pulmonary and extra pulmonary tuberculosis for relevant studies was updated in May 2010 and WHO has performed a bivariate meta-analysis that jointly modeled both test sensitivity and specificity (http://www.who.int/tb/laboratory/policy_statements/en/index.html). It has been concluded that the commercially available serological tests provide inconsistent and imprecise findings and the sensitivity and specificity of the tests were highly variable. Our approach presented in this study, has not only identified a large number of proteins unique to *M. tuberculosis*, but in parallel provided the information on the coverage. The higher the coverage, higher is the abundance of the protein in the PPD sample analyzed. Our results are correlating with earlier publications and many of the proteins identified in the PPD of *M. tuberculosis*, have already been analyzed for their potential as a diagnostic markers. Some of the hits we have found are GroES [52,53], GroEL [54,55], protein EsxB (EsxB) [56] heat shock protein HspX (HspX) [57], hypothetical protein (TB15.3), hypothetical protien (TB16.3) [58], 50S ribosomal protein L7/L12 (RplL) [59], hypothetical protein (EsxA) [60], immunogenic protein Mpt63 (Mpt63) [61], Mpt64 [62], ESAT-6 like protein EsxJ (EsxJ), and ESAT-6 like protein EsxO (EsxO) [63]. Based on these observations, many more *M. tuberculosis* PPD can be analyzed and the abundant antigens be evaluated for their potential as diagnostics biomarkers.

### Clinical applications of the study

Based on our findings, out of 265 proteins identified in PPD-CT68, 142 proteins were found common between PPD-CT68 and PPD-S2. The common proteins can further be evaluated for their potential as skin test antigens. Proteins identified in our analysis, which are absent in *M. avium* and *M. bovis* and do not show any significant identity with proteins from lung infecting bacteria can be shortlisted for developing various immunological assays to identify *M. tuberculosis*, on the basis of their seroreactivity and abundance. For example, Rv2346, Rv0379 and Rv1388 were found to be absent in PPD *M. bovis* and PPD *M. avium* and possessed least identity with proteins from lung infecting pathogens. As discussed earlier, one of the major issues with the use of PPD as a skin test antigen is false positive results for the individuals with BCG vaccination. Use of antigens absent in *M. bovis* may help overcoming that. Global profiling of antigens in PPD may help to identify *M. tuberculosis*-specific antigens, which are not present in BCG. These antigens will be useful in differentiating infected from vaccinated individuals. PPD can be prepared from *M. tuberculosis, M. avium* and *M. bovis*, so if we can use antigen specific to every strain to elicit the test response we can diagnose the species of *Mycobacteria* with a simple skin test. Subset of antigens that is mainly responsible for activating the immune response can be used in adjunction with BCG or for booster doses to enhance immune response. By knowing the antigens involved in the test response, we can use minimal essential amount of PPD for TST. Use of specific antigens in TST will make it more specific and will reduce the false positive results due to antigen cross reaction.

## Conclusions

Despite the identification of almost a dozen antigens for developing next generation PPD, it is challenging to replace the classical PPD preparation. ESAT-6, Mpt64, recombinant antigen (DPPD), CFP10, recombinant truncated 38 kDa protein (TPA38), DnaK, GroEL, RplL are currently under evaluation as next generation PPD candidates [9,29,62,64-72]. PPD is a crude extract obtained after several steps of filtration, purification, and precipitation with trichloroacetic acid [30]. Enough knowledge on PPD composition and contribution of individual antigens in TST would give a better insight to understand the molecular mechanism behind it and will also allow the researchers to select a combination of proteins specific to *M. tuberculosis*. Our analysis further revealed mycobacterial proteins in PPD-CT68 sharing identical amino acid sequence with lung infecting bacteria. Detailed epitopic analysis of these proteins may help the researchers to understand the mechanism behind cross reactivity in TST.

Mass Spectrometry is an efficient tool for proteomic analysis due to its high mass accuracy, sensitivity and ability to deal with complex sample mixtures. Here, in this study we used a high resolution Fourier transform mass spectrometer for LC-MS/MS analysis of PPD-CT68. Many of the proteins identified in the PPD of *M. tuberculosis*, have already been analyzed for their potential as diagnostic markers. The complete protein profile of PPD-CT68 uncovered from this study can be used to analyze immune response and antibody production pattern of body against different PPD antigens.

### Data availability

The mass spectrometry proteomics data have been deposited to the ProteomeXchange Consortium (http://proteomecentral.proteomexchange.org) via the PRIDE partner repository [73] with the dataset identifier PXD000377.

## Methods

### Trypsin digestion

PPD-CT68 (100 μg protein) was subjected to in-solution digestion using trypsin. Reduction and alkylation were carried out using 5 mM dithiothreitol (60°C for 45 min) and 20 mM iodoacetamide (RT for 10 min), respectively. Overnight digestion was carried out using trypsin with an enzyme: substrate ratio of 1:20 at 37°C [74]. The digest was then purified using sep-pak C18 columns (WAT051910, Waters Corporation, MA) [75] and the eluates were lyophilized at -52°C (Operon, Gyeonggi-do, Korea) and stored at -80°C until LC-MS/MS analysis.

### Mass spectrometry

We have carried out the LC-MS/MS analysis on an LTQ-Orbitrap Velos ETD mass spectrometer (Thermo Scientific, Bremen, Germany) interfaced with an Agilent 1100 HPLC system (Agilent Technologies, Santa Clara, CA). Trypsin digested PPD peptides were analyzed on a reversed phase liquid chromatography. The RP-LC system equipped with a pre-column (2 cm, 5 μ – 100A°), analytical column (10 cm, 5 μ – 100A°) made with magic AQ C_18_ material (PM5/61100/00; Bruker-Michrom Inc., Auburn, CA) packed in-house. Further, the peptides were sprayed using an electro-spray emitter tip 8 μ (New Objective, Woburn, MA) fixed to a nanospray ionization source. The peptides were loaded on the pre-column using 97% solvent A (0.1% formic acid (aq) and resolved on the analytical column using a gradient of 10-30% solvent B (90% acetonitrile, 0.1% formic acid) for 60 min at a constant flow rate of 0.35 μl/min. The spray voltage and heated capillary temperature were set to 2.0 kV and 220°C, respectively. The data acquisition was performed in a data dependent manner. From each MS survey scan, 10 most intense precursor ions were selected for fragmentation. MS and MS/MS scans were acquired in an Orbitrap mass analyzer and the peptides were fragmented by higher energy collision dissociation with normalized collision energy of 39%. MS scans were acquired at a resolution of 60,000 at 400 m/z, while MS/MS scans were acquired at a resolution of 15,000. The automatic gain control for full FT MS was set to 0.5 million ions and for FT MS/MS was set to 0.1 million ions with maximum time of accumulation of 750 ms and 100 ms, respectively. The raw data obtained was submitted to ProteomeXchange Consortium (http://proteomecentral.proteomexchange.org).

### Data analysis

We searched the MS/MS data using Sequest search algorithm on Proteome Discoverer (version 1.3.0.339, Thermo Scientific, Bremen, Germany), against protein database of *M. tuberculosis* H37Rv strain downloaded from NCBI RefSeq (updated December 29, 2011). The search parameters are: a) precursor mass range between 350 to 8000 Da; b) minimum peak count was set to 5; c) signal to noise threshold set to 1.5; d) trypsin was used as a proteolytic enzyme allowing up to one missed cleavage; e) precursor mass tolerance of 20 ppm and fragment tolerance of 0.1 Da; f) oxidation of methionine as variable modification and carbamidomethylation of cysteine as fixed modification; and g) 1% false discovery rate (FDR).

## Abbreviations

AcpP: Acyl carrier protein; Apa: Alanine and proline rich protein; BCG: Bacillus Calmette-Guerin; BfrA: Bacterioferritin; BLAST: Basic Local Alignment Search Tool; CFP10: Culture filtrate antigen EsxB; EF-Tu: Elongation factor 2; EsxJ: ESAT-6 like protein EsxJ; EsxO: ESAT-6 like protein EsxO; ESAT-6: Early secretory antigenic target-6; FbpA: Secreted antigen 85A; GroES: Co-chaperonin GroES; HspX: Heat shock protein HspX; IGRA: Interferon gamma release assay; IP-10: Interferon gamma induced protein 10; LC-MS/MS: Liquid chromatography-mass spectrometry; LprG: Lipoprotein LprG; LTBI: Latent tuberculosis infection; Mpt83: Cell surface lipoprotein Mpt83; PepA: Serine protease PepA; PPD-CT68: Purified protein derivative-Connaught tuberculin 68; RpiL: 50S ribosomal protein L7/L12; SahH: S-adenosylhomocysteine hydrolase; Tpx: Thiol peroxidase; TrxC: Thioredoxin; TPA38: Truncated 38kDa protein; TST: Tuberculin skin test.

## Competing interests

The authors declare that they have no competing interests.

## Authors’ contribution

JN, TSKP and AP conceived the study and designed the experiments; SK, NRS, GJS and HCH carried out the experiments; TSKP, HCH, RV, AKM and JS analyzed the data; TSKP, RV and VNP wrote the manuscript; JN, AG, AC and VPM provided critical comments. All authors read and approved the final manuscript.

## Supplementary Material

Additional file 1: Table S1 List of peptides identified from M. tuberculosis PPD (CT68). Click here for file

Additional file 2: Table S2 List of proteins identified from M. tuberculosis PPD (CT68). Click here for file

Additional file 3: Table S3a List of Mycobacterium tuberculosis PPD-CT68 peptides with 10 or more amino acid identity with 24 lung infecting bacteria. **Table S3b. **List of Mycobacterium tuberculosis proteins corresponding to the peptides with 10 or more amino acid identity with 24 lung infecting bacteria. **Table S3c. **List of Mycobacterium tuberculosis proteins with sequence identity of ≥ 20 amino acid identity with 24 lung infecting bacteria. Click here for file
